# Elevated NKT cell levels in adults with severe chronic immune thrombocytopenia

**DOI:** 10.3892/etm.2013.1386

**Published:** 2013-11-06

**Authors:** RUILONG XU, ZHAOJING ZHENG, YONGJUN MA, YINGPING HU, SHUNHONG ZHUANG, BIN WEI, JIANXING LU

**Affiliations:** 1 Key Laboratory of Laboratory Medicine, Ministry of Education, Zhejiang Provincial Key Laboratory of Medical Genetics, Wenzhou Medical University, Wenzhou, Zhejiang 325035, P.R. China; 2 Department of Laboratory Medicine, Jinhua Hospital of Zhejiang University, Jinhua, Zhejiang 321000, P.R. China; 3 Department of Hematology, Jinhua Hospital of Zhejiang University, Jinhua, Zhejiang 321000, P.R. China

**Keywords:** chronic immune thrombocytopenia, natural killer T cells, regulatory T cells, cytokine profile, adult

## Abstract

The aim of this study was to investigate the frequency of circulating natural killer T (NKT) cells and regulatory T cells (Tregs), as well as serum cytokine profiles, in adult chronic primary immune thrombocytopenia (ITP). The frequency of circulating T cell receptor (TCR) Vα24^+^Vβ11^+^CD3^+^ NKT cells and CD4^+^CD25^+^CD127^−/low^ Tregs was measured using multi-color flow cytometry. The serum concentrations of 11 cytokines were determined with a cytometric bead assay. The frequency of circulating NKT cells in patients with ITP was 0.13±0.03%, whereas the frequency in healthy controls was 0.07±0.01% of CD3^+^ (P>0.05). However, the frequency of NKT cells in patients with ITP with platelet counts ≤20×10^9^/l (0.22±0.05%) was significantly higher than that in patients with platelet counts >20×10^9^/l (0.05±0.01%; P<0.05) and that in healthy controls (0.07±0.01%; P<0.05). The frequency of peripheral Tregs was comparable between patients with ITP (3.97±0.44% of CD4^+^) and healthy controls (3.69±0.31%; P>0.05). No significant differences were observed in the serum concentrations of 11 cytokines between patients with ITP and healthy controls, despite the fact that the serum levels of interleukin (IL)-12p70, IL-8, IL-4, interferon (IFN)-γ and tumor necrosis factor (TNF)-α in patients with ITP were higher than those in the healthy controls. The platelet count was negatively correlated with the frequency of circulating NKT cells in chronic ITP. These results indicate that NKT cells may be involved in ITP with severe thrombocytopenia, and NKT and Tregs may be important in cytokine deregulation in chronic ITP.

## Introduction

Primary immune thrombocytopenia (ITP) is an acquired immune-mediated disorder characterized by isolated thrombocytopenia, which is defined as a peripheral blood platelet count of <100×10^9^/l and the absence of any clear initiating and/or underlying cause (1). Antibodies that are autoreactive to platelet antigens, mainly the platelet glycoprotein IIb/IIIa complex, are considered responsible for the reduced platelet production and accelerated destruction of platelets by the reticuloendothelial system (2,3). Platelet antigen-specific T cells are activated upon the recognition of platelet auto-antigens and induce the production of auto-antibodies by B cells in patients with ITP (4,5). ITP has been further suggested to be a T helper (Th)-1-polarized autoimmune disease (6–8). These data are consistent with a loss of peripheral tolerance and an inflammatory phenotype in patients with chronic ITP.

CD4^+^CD25^+^ regulatory T cells (Tregs) are critical in the maintenance of peripheral tolerance and directly and indirectly suppress the activation and proliferation of numerous cell types, including T, B, dendritic, natural killer and natural killer T (NKT) cells *in vivo* and/or *in vitro*(9). A number of studies have been conducted to investigate the roles of Tregs in ITP, particularly in chronic ITP; however, the results have not always been consistent (10–12). Liu *et al*(10) and Sakakura *et al*(11) observed that the level of Tregs was significantly decreased in the circulation in ITP. By contrast, Yu *et al*(12) demonstrated that the level of circulating Tregs was comparable between patients with ITP and the controls; however, the inhibitory activity of the Tregs isolated from the patients with ITP was two-fold lower than that of the Tregs from the controls.

NKT cells are another T lymphocyte subset with regulatory functions involved in peripheral tolerance in humans, and are characterized by invariant expression of the T cell receptor (TCR) Vα24 and Vβ11 chains (13). The levels and functional status of NKT cells are associated with multiple human autoimmune diseases; however, the mechanisms have yet to be elucidated (14). Johansson *et al*(15) demonstrated that the levels of circulating NKT cells decreased in patients with ITP, which suggested the involvement of NKT cells in ITP pathogenesis.

NKT cells and Tregs interact with each other and contribute functionally to a sophisticated network of immune regulation in humans (16). However, few studies have described the changes in levels of circulating Tregs and NKT cells in ITP. In this study, the frequency of peripheral Tregs and NKT cells and the Th1/Th2 cytokine profile were analyzed, and a correlation analysis was performed between the immune response and the disease phenotypes in adult chronic ITP.

## Materials and methods

### Subjects

Sixty-eight patients with chronic ITP, who were hospitalized in the Department of Hematology, Jinhua Hospital of Zhejiang University (Jinhua, China) from January 2008 to March 2010, were included in this study. An additional 38 healthy age- and gender-matched volunteers were used as controls. Gender, mean age and platelet count from the ITP and control groups are summarized in Table I. The diagnosis of chronic ITP was in agreement with the standards proposed by Zhang *et al*(17), i.e., a platelet count of <50×10^9^/l for more than six months, normal or increased bone marrow, megakaryocytes without any features of dysplasia and a lack of other known causes, such as systemic lupus erythematosus. In the two weeks prior to sampling, none of the patients or healthy volunteers took corticosteroids or other medications that may have affected platelet metabolism. All patients were in the active phase and were divided into two groups according to platelet count: <20×10^9^/l (n=30) and >20×10^9^/l (n=38). This study was conducted in accordance with the Declaration of Helsinki and with approval from the ethics committees of Jinhua Hospital of Zhejiang University and Wenzhou Medical University (Wenzhou, China). Written informed consent was obtained from all participants.

### Preparation of peripheral blood mononuclear cells (PBMCs)

EDTA-K_2_ anti-coagulated venous blood, collected from patients and healthy volunteers, was diluted with an equivalent volume of saline. According to standard procedures, PBMCs were separated over Lymphoprep™ 1.077 medium (Axis-Shield PoC, Oslo, Norway), washed three times and suspended in pH 7.4 phosphate-buffered saline (PBS) with an adjusted cell density of 5×10^6^/ml.

### Measurement of CD4^+^CD25^+^CD127^−/low^ cells

PBMCs were stained with a cocktail of fluorescein isothiocyanate (FITC) anti-human CD4, phycoerythrin (PE) anti-human CD25, CD127-PE-Cy5 and immunoglobulin (Ig) G1-PE-Cy5 (all from eBioscience, Inc., San Diego, CA, USA) for 15 min at room temperature in the dark, washed three times with pH 7.4 PBS and resuspended in PBS. The frequency of CD4^+^CD25^+^CD127^−/low^ cells was determined by three-color flow cytometry on a Beckman-Coulter Epics XL flow cytometer (Beckman-Coulter, Inc., Brea, CA, USA). Flow-Check™ fluorospheres (Beckman-Coulter, Inc.) were used in the daily alignment and verification of the flow cytometer optics and fluidics. Data acquisition and analysis were performed using the Expo32 ADC software package (Applied Cytometry, Dinnington, UK). Tregs were identified as CD4^+^CD25^+^CD127^−/low^ and the frequency of Tregs was expressed as the percentage of CD4^+^ cells. A total of ≥70,000 CD4^+^ events were analyzed in each experiment.

### Measurement of TCRVα24^+^Vβ11^+^ T cells

PBMCs were stained with CD3-PE-Cy5, Vα24-FITC and Vβ11-PE (all from Beckman-Coulter, Inc.) for 15 min at room temperature in the dark, washed three times with pH 7.4 PBS and resuspended in PBS. Simultaneously, isotype controls were set with IgG1-FITC and IgG2a-PE (Beckman-Coulter, Inc.). NKT cells were identified as CD3^+^Vα24^+^Vβ11^+^ and the frequency of NKT cells was expressed as the percentage of CD3^+^ cells. A total of ≥100,000 CD3^+^ events were analyzed in each experiment.

### Th1/Th2 cytokine profiling

Serum was collected from each patient and healthy volunteer and stored in a −76°C ultra-low temperature freezer (Thermo Fisher Scientific, Inc., Middletown, VA, USA) until analysis. Serum Th1/Th2 cytokine profiles were determined using a cytometric bead array (CBA) Human Th1/Th2 Cytokine 11-plex kit (eBioscience, Campus Vienna-Biocenter 2, Vienna, Austria) and 11 cytokines, interleukin (IL)-12p70, IL-10, IL-2, IL-8, IL-6, IL-5, IL-4, IL-1β, interferon (IFN)-γ, tumor necrosis factor (TNF)-α and TNF-β, were analyzed. The experimental procedures were in strict accordance with the manufacturer’s instructions. Data were acquired through the Expo32 ADC software package (Applied Cytometry) on a Beckman-Coulter Epics XL flow cytometer (Beckman-Coulter, Inc.). Data analysis was performed using FlowCytomix™ Pro 2.3 (eBioscience, Campus Vienna-Biocenter 2). The concentration of serum cytokines was expressed in pg/ml.

### Statistical analysis

Data were processed statistically using SPSS 16.0 (SPSS, Inc., Chicago, IL, USA) and GraphPad Prism 4.0 (GraphPad Software, Inc., La Jolla, CA, USA) software. A Student’s t-test was used to compare two independent samples, while one-way analysis of variance (ANOVA) was used for multiple comparisons. A least significant difference (LSD) test was performed for comparisons in which equal variances were assumed and Dunnett’s T3 test was used for comparisons in which equal variances were not assumed. Non-parametric comparisons were conducted using Pearson’s χ^2^ test and a linear regression model was used for correlation analysis. P<0.05 was considered to indicate a statistically significant difference.

## Results

### Measurement of NKT cells

**Figure 1:**
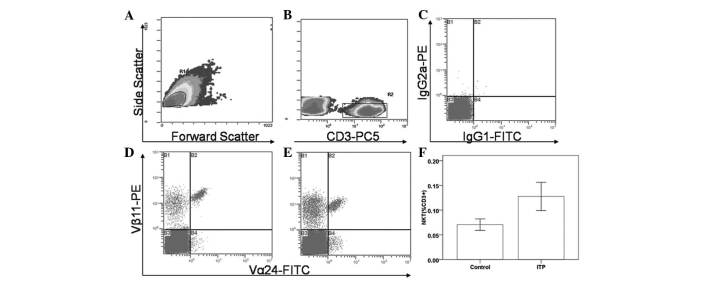
Dot plot of natural killer T (NKT) cells in peripheral blood from patients with immune thrombocytopenia (ITP) and healthy controls by flow cytometry. CD3^+^ cells from peripheral blood mononuclear cells (PBMCs), gated on (A) scatter signals and (B) SSC/CD3, were tested for expression of Vα24 and Vβ11. (C) The threshold of positivity for Vα24 and Vβ11 was set with isotype controls. The distributions of NKT cells (CD3^+^Vα24^+^Vβ11^+^), expressed as the percentage of CD3^+^ cells, from (D) adults with chronic ITP and (E) healthy controls were demonstrated and (F) compared. Ig, immunoglobulin; FITC, fluorescein isothiocyanate; PE, phycoerythrin.

The level of NKT cells (Fig. 1) in patients with chronic ITP was higher than that in the controls (0.13±0.03 versus 0.07±0.01% of CD3^+^); however, the difference was not statistically significant (P>0.05). The results showed that the frequency of NKT cells was significantly elevated in patients with platelet counts ≤20×10^9^/l (0.22±0.05%) compared with the frequency of NKT cells in patients with platelet counts >20×10^9^/l (0.05±0.01%; P<0.05) and in controls (0.07±0.01%; P<0.05); however, no significant difference was observed between the latter two groups.

### Frequency of Tregs

**Figure 2:**
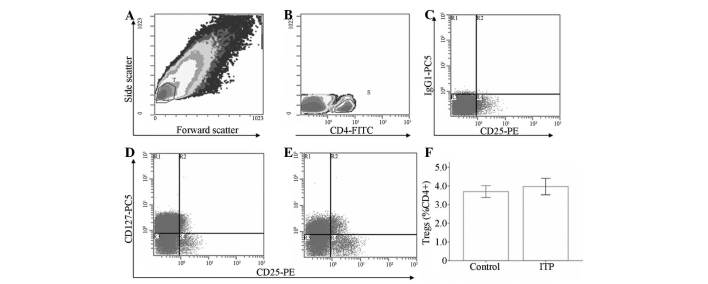
Dot plot of regulatory T cells (Tregs) in peripheral blood from patients with immune thrombocytopenia (ITP) and healthy controls. CD4^+^ cells from peripheral blood mononuclear cells (PBMCs), gated on (A) scatter signals and (B) SSC/CD4, were tested for expression of CD25 and CD127. (C) The threshold of positivity for CD25 and CD127 was set with isotype controls. The distributions of Treg cells (CD4^+^CD25^+^CD127^−/low^), expressed as the percentage of CD4^+^ cells, from (D) adults with chronic ITP and (E) healthy controls were demonstrated and (F) compared. FITC, fluorescein isothiocyanate; Ig, immunoglobulin; PE, phycoerythrin.

The frequency of circulating Tregs (Fig. 2) in patients with chronic ITP was 3.97±0.44% of CD4^+^, which was comparable to 3.69±0.31% in the control group (P>0.05). Compared with Treg level in the patients with platelet counts >20×10^9^/l (3.78±0.59%), the level of Tregs was elevated in patients with chronic ITP with platelet counts ≤20×10^9^/l (4.21±0.67%); however, the difference was not statistically significant (P>0.05).

### Th1/Th2 cytokine ratio

**Figure 3:**
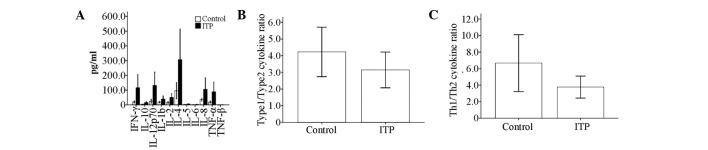
T helper (Th)-1/Th2 cytokine profile in serum from patients with immune thrombocytopenia (ITP) and healthy controls, as determined by cytometric bead array (CBA). (A) Serum levels of interleukin (IL)-12p70, IL-10, IL-2, IL-8, IL-6, IL-5, IL-4, IL-1β, interferon (IFN)-γ, tumor necrosis factor (TNF)-α and TNF-β were compared between adults with chronic ITP and healthy controls. (B) Relative expression of type 1 cytokines (IFN-γ, IL-2, IL-12p70 and TNF-β) versus type 2 cytokines (IL-4, IL-5, IL-6 and IL-10). (C) Th1 (IFN-γ and IL-2) and Th2 cytokines (IL-4 and IL-5) were compared.

**Figure 4:**
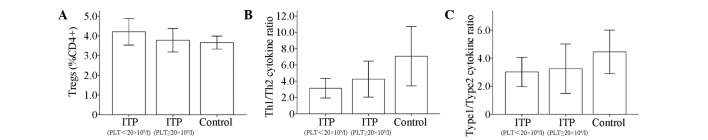
Frequency of peripheral regulatory T cells (Tregs) in patients with immune thrombocytopenia (ITP) and controls. (A) Treg frequency in adults with chronic ITP with platelet counts (PLTs) of ≤20×10^9^/l and >20×10^9^/l and in healthy controls. (B) T helper (Th)-1/Th2 cytokine expression ratio. (C) Type 1/type 2 cytokine expression ratio.

No significant differences were observed in the serum levels of IL-12p70, IL-10, IL-2, IL-8, IL-6, IL-5, IL-4, IL-1β, IFN-γ, TNF-α or TNF-β between the patients with ITP and the controls (Table II). The Th1 cytokine (IFN-γ, IL-2)/Th2 cytokine (IL-4, IL-5) ratio was calculated, which was used to predict the disease-specific Th cell polarization. The type 1 cytokine (IFN-γ, IL-2, IL-12p70 and TNF-β)/type 2 cytokine (IL-4, IL-5, IL-10 and IL-6) ratio was also calculated, which was used to evaluate the host’s overall immune response. The results showed that the Th1/Th2 ratios in patients with ITP and the controls were 3.77±1.34 and 6.67±3.45, respectively, which indicated a relative Th2 polarization in patients with ITP compared with the controls. However, the difference did not reach statistical significance (P>0.05). In addition, a similar trend was observed in the type 1/type 2 ratio between patients with ITP and controls, with ratios of 3.14±1.07 and 4.23±1.48, respectively (P>0.05; Table II, Fig. 3). Furthermore, no significant difference was observed in either the Th1/Th2 ratio or the type 1/type 2 ratio between patients with ITP with platelet counts >20×10^9^/l and patients with platelet counts ≤20×10^9^/l or the controls (Table III, Fig. 4).

### Negative correlation between circulating NKT cells and platelet count

**Figure 5:**
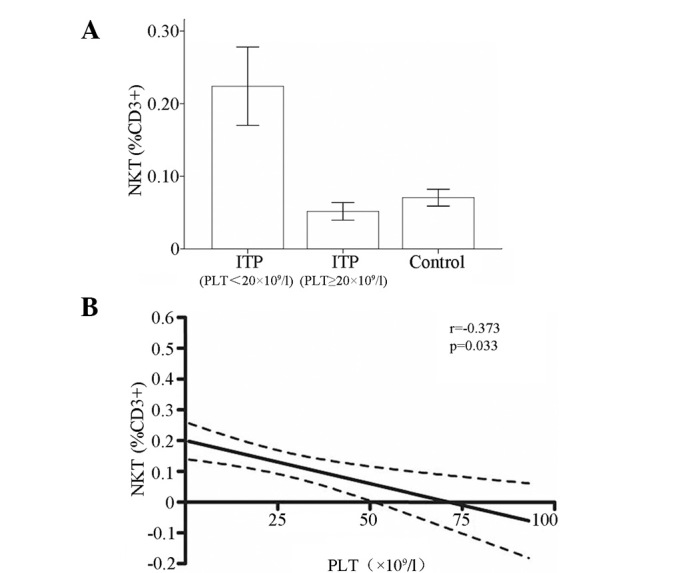
Frequency of circulating natural killer T (NKT) cells and the correlation with platelet count (PLT) in chronic immune thrombocytopenia (ITP). (A) Comparison of peripheral frequency of NKT cells between groups of adults with chronic ITP with PLT ≤20×10^9^/l, patients with PLT >20×10^9^/l and healthy controls. (B) Frequency of NKT cells versus peripheral blood platelet concentration linear regression line (solid) with 95% confidence intervals (dotted lines) in patients with ITP (r=-0.373; P=0.033).

Although the difference in the frequency of circulating NKT cells between the patients with ITP and the controls was marginal, the level of NKT cells in patients with chronic ITP and severe thrombocytopenia (≤20×10^9^/l) was significantly elevated compared with that in either the controls or the patients with moderate thrombocytopenia (>20×10^9^/l). Thus, a correlation analysis was performed between the level of circulating NKT cells and the platelet count in patients with ITP. A negative correlation between platelet count and NKT cell circulation level was revealed by a linear regression analysis in adult patients with chronic ITP (r=−0.373; P=0.033; Fig. 5). In addition, a positive correlation between the frequency of Tregs and the Th1/Th2 ratio was detected in adults with chronic ITP (r=0.451; P = 0.011). Also, the platelet count was positively correlated with serum levels of IL-12p70 (r=0.354; P=0.044), IFN-γ (r=0.365; P=0.037), IL-4 (r=0.354; P=0.044) and TNF-α (r=0.366; P=0.036) in patients with ITP (data not shown). However, the results did not reveal a correlation between circulating Tregs and peripheral NKT cells in adult chronic ITP.

## Discussion

NKT cells and Tregs are important in the maintenance of peripheral tolerance in humans. Abnormalities in the levels or quality of NKT cells and Tregs have been implicated in numerous autoimmune diseases. The loss of peripheral tolerance of a host immune system to platelet auto-antigens leads to premature platelet destruction and a variety of clinical presentations in patients with ITP. Liu *et al*(10) and Sakakura *et al*(11) observed that levels of circulating Tregs decreased significantly in patients with ITP. However, Yu *et al*(12) demonstrated that the inhibitory activity, and not the number, of Tregs in the peripheral blood of patients with ITP contributed to the loss of peripheral tolerance in ITP. By contrast, our results showed that levels of circulating Tregs were not decreased significantly in adult chronic ITP, unlike in the controls. This discrepancy may have been due to the different protocols for the identification of Tregs in different studies. Transcription factor FoxP3 has been indicated to be the most efficacious marker for Treg identification to date (18). However, FoxP3 expression was also detected in CD4^+^ cells with low or negative CD25 antigen and CD8^+^ cells (18), thereby leading to an inaccurate measurement of Tregs. Liu *et al*(19) revealed that a phenotype of CD4^+^CD25^+^CD127^+/−^ was able to be used reliably as a marker for functional Tregs in humans. Different markers for the identification of Tregs may, to some extent, explain the discrepancy in the measurements of Tregs between different studies, including the present study.

Johansson *et al*(15) identified that the proliferative potential of peripheral NKT cells was markedly decreased in patients with ITP compared with the control group. Furthermore, the decreased proliferative potential of NKT cells worsened following corticosteroid therapy, which indicated that NKT cells are involved in ITP pathogenesis. Levels of NKT cells were noted to be elevated in the peripheral blood of a female with ITP; these elevated NKT cells inhibited the *in vitro* proliferation of autologous CD4^+^ T cells, which indicated the protective role of NKT cells in ITP (20). This observation was further supported by the results of Ho *et al*(21), which indicated that activated NKT cells inhibited the *in vitro* proliferation of CD8^+^ cells, and that CD8^+^ NKT cells suppressed T cell activation through the killing mechanism of antigen-presenting cells. In the present study, the levels of circulating NKT cells increased in patients with ITP; however, the difference between the patients with ITP and the controls was not statistically significant. Further analysis revealed that NKT cell levels were markedly elevated in adult patients with ITP with severe thrombocytopenia, which suggested the importance of NKT cells in ITP, particularly with severe thrombocytopenia. In addition, our results showed a negative correlation between platelet count and peripheral NKT cells in ITP. Our results and data from other studies further indicate that NKT cells are important in the pathogenesis of ITP. Cao *et al*(22) observed that plasma levels of IL-22 were significantly increased in patients with active ITP, and high-dose dexamethasone administration reduced IL-22 production and corrected the imbalance between Th1 and Th22 subsets. The NKT cell is an IL-22-secreting cell *in vivo*(23). Thus, IL-22 and NKT cells in chronic ITP appear to be correlated.

Azuma *et al*(24) reported that Tregs inhibited the proliferation, cytokine secretion (including IFN-γ, IL-4, IL-13 and IL-10) and cytotoxicity of CD4^+^ and CD4^−^CD8^−^ NKT cells through a cell-cell contact mechanism. NKT cells, particularly CD4^+^ NKT cells, promoted Treg proliferation through IL-2 synthesis and secretion (25). However, in the present study, no correlation between NKT cells and Tregs was identified. Additional studies are required to delineate the correlation between these two regulatory T cell subsets in chronic ITP.

In this study, no significant difference was observed in the serum cytokine profiles and the Th1/Th2 and type 1/type 2 ratios between the patients with ITP and the controls. Our data contradicted the results of other studies on serum cytokine profiles in ITP (6,8). However, linear regression analysis showed that the platelet count correlated positively with the serum levels of IL-12p70, IFN-γ, IL-4 and TNF-α in patients with ITP, which implied the involvement of cytokines in ITP.

At present, a direct comparison between different studies is difficult due to the exclusive nature of the ITP diagnosis (26) and the diversity of the disease phenotype. Zehnder *et al*(27) proposed that a specific labeling and reliable enumeration for platelet antigen-specific T and B cells was necessary in the future. Accordingly, investigations into ITP may be expanded considerably when platelet antigen-specific T and B cell data are combined with the analysis of NKT cells, Tregs and serum cytokine profiles in adult chronic ITP.

## Figures and Tables

**Table I:** Summary of the clinical and laboratory parameters of the study subjects.

Group	Male/female (n/n)	Age (years)	PLT (x10^9^/l)
ITP	40/28	43.82±2.46	26.69±3.90
Control	24/14	38.53±1.97	230.84±11.29

Results are presented as the mean ± standard deviation. ITP, immune thrombocytopenia; PLT, platelet count.

**Table II:** Results of serum cytokine profiling in patients with ITP and controls.

	Cytokine level (pg/ml)		
		
Cytokine	ITP	Control	P-value
IFN-γ	116.38±88.79	20.03±7.35	>0.05
IL-10	16.05±7.03	4.20±0.95	>0.05
IL-12p70	131.92±90.74	24.59±13.96	>0.05
IL-1β	39.47±20.96	18.94±5.67	>0.05
IL-2	51.12±24.04	16.13±6.54	>0.05
IL-4	306.84±207.59	96.27±54.69	>0.05
IL-5	5.52±2.26	2.19±0.73	>0.05
IL-6	2.66±1.75	1.36±1.00	>0.05
IL-8	105.59±77.24	36.08±9.92	>0.05
TNF-α	88.68±65.01	18.65±6.73	>0.05
TNF-β	0.60±0.60	0.00±0.00	>0.05

Results are the mean ± standard deviation. ITP, immune thrombocytopenia; IFN, interferon; IL, interleukin; TNF, tumor necrosis factor.

**Table III:** Results of serum cytokine profiling in patients with ITP with severe (PLT ≤20×10^9^/l) and moderate (PLT >20×10^9^/l) thrombocytopenia and controls.

	Cytokine level (pg/ml)			
		
Cytokine	ITP (PLT ≤20×10^9^/l)	ITP (PLT >20×10^9^/l)	Control	P-value
IFN-γ	17.64±9.29	194.34±158.25	20.03±7.35	>0.05
IL-10	16.67±11.94	15.56±8.64	4.20±0.95	>0.05
IL-12p70	22.01±12.39	218.68±161.15	24.59±13.96	>0.05
IL-1β	27.02±21.62	49.29±33.79	18.94±5.67	>0.05
IL-2	39.59±19.66	60.23±40.59	16.13±6.54	>0.05
IL-4	64.34±48.44	498.29±367.87	96.27±54.69	>0.05
IL-5	6.52±4.43	4.72±2.14	2.19±0.73	>0.05
IL-6	0.94±0.66	4.02±3.09	1.36±1.00	>0.05
IL-8	45.02±31.92	153.42±136.59	36.08±9.92	>0.05
TNF-α	20.32±9.29	142.64±115.96	18.65±6.73	>0.05
TNF-β	1.37±1.37	0.00±0.00	0.00±0.00	>0.05

Results are the mean ± standard deviation. ITP, immune thrombocytopenia; PLT, platelet count; IFN, interferon; IL, interleukin; TNF, tumor necrosis factor.
